# Sanctions as honest signals – The evolution of pool punishment by public sanctioning institutions

**DOI:** 10.1016/j.jtbi.2014.04.019

**Published:** 2014-09-07

**Authors:** Sarah Schoenmakers, Christian Hilbe, Bernd Blasius, Arne Traulsen

**Affiliations:** a University of Oldenburg, Carl-von-Ossietzky-Straße 9-11, 26111 Oldenburg, Germany; b Program for Evolutionary Dynamics, Harvard University, One Brattle Square, Cambridge, MA 02138, USA; c Department for Evolutionary Theory, Max Planck Institute for Evolutionary Biology, August-Thienemannstraße 2, 24306 Plön, Germany

**Keywords:** Evolution of cooperation, Public goods game, Institution formation, Tragedy of the commons

## Abstract

In many species, mutual cooperation is stabilized by forms of policing and peer punishment: if cheaters are punished, there is a strong selective pressure to cooperate. Most human societies have complemented, and sometimes even replaced, such peer punishment mechanisms with pool punishment, where punishment is outsourced to central institutions such as the police. Even before free-riding occurs, such institutions require investments, which could serve as costly signals. Here, we show with a game theoretical model that this signaling effect in turn can be crucial for the evolution of punishment institutions: In the absence of such signals, pool punishment is only stable with second-order punishment and can only evolve when individuals have the freedom not to take part in any interaction. With such signals, individuals can opportunistically adjust their behavior, which promotes the evolution of stable pool punishment even in situations where no one can stand aside. Thus, the human propensity to react opportunistically to credible punishment threats is often sufficient to establish stable punishment institutions and to maintain high levels of cooperation.

## Highlights

• We show how public knowledge about punishment institutions can affect their evolution.
• Subjects can establish a punishment institution before they play a public goods game.
• The evolutionary dynamics leads to a coexistence of pool punishers and opportunists.
• Pool punishment can evolve without second-order punishment and without loners.

## 1 Introduction

When individuals share common resources, or when they engage in mutualistic relationships, they may be tempted to reap the benefits without paying the associated costs (Hardin, 1968; Trivers, 1971). Stable cooperation thus requires mechanisms that suppress selfish behavior (Nowak, 2006; Sigmund, 2010). One such mechanism is punishment: various biological examples suggest that sanctions can create a sufficiently strong selective pressure to prevent cheating (Clutton-Brock and Parker, 1995; Sigmund, 2007). Sanctions can take different forms, such as the selective abortion of overexploited flowers in plant–pollinator mutualisms (Goto et al., 2010), or the chasing of cheating cleaner fishes (Bshary and Grutter, 2005). In human societies, sanctions may range from merely symbolic acts or monetary fines, to imprisonment and the exclusion of individuals from the community (Guala, 2012; Sasaki and Uchida, 2013). In all these instances, punishment is thought to prevent cheating as it creates additional incentives for individuals to cooperate.

From a theoretical viewpoint, there are two different ways to implement a punishment regime (Sigmund et al., 2010). One way is to rely on decentralized sanctions, such that cheaters are directly punished by their victims or by present third parties (e.g. Raihani et al., 2010). Several models have shown how such a system of peer punishment can evolve due to the effects of group selection (Boyd et al., 2003), optional participation (Fowler, 2005; Hauert et al., 2007, 2008; De Silva et al., 2010), local interactions (Nakamaru and Iwasa, 2006; Helbing et al., 2010a,b), and coordination (Boyd et al., 2010). However, especially for humans, the effect of decentralized sanctions on overall welfare remains disputed, as the use of punishment may actually decrease the overall welfare (Dreber et al., 2008; Gächter et al., 2008). Moreover, several studies have revealed detrimental sides of peer punishment, as it may provoke anti-social punishment (Herrmann et al., 2008; Rand and Nowak, 2011; Powers et al., 2012), or counter-punishment (Nikiforakis, 2008; Janssen and Bushman, 2008; Fehl et al., 2012). Although these problems can be circumvented when individuals have the option to abstain from the public good such that they cannot be punished (García and Traulsen, 2012), or when the individuals׳ reputation is at stake (Sigmund et al., 2001; Hilbe and Sigmund, 2010; dos Santos et al., 2011; Hilbe and Traulsen, 2012), peer punishment is often considered as a form of self-justice, which is not desirable as it can be misused.

In view of these shortcomings, many human societies have delegated the prosecution of wrong-doers to central sanctioning institutions (Sigmund et al., 2010, 2011; Baldassari and Grossman, 2011; Sasaki et al., 2012; Cressman et al., 2012; Perc, 2012; Traulsen et al., 2012; Zhang et al., 2013; Hilbe et al., 2014). Maintaining such pool punishment institutions is costly and requires financial contributions in the form of taxes and fees, ideally paid by all beneficiaries. Such a regime has the apparent disadvantage that the costs of pool-punishment arise even when there are no exploiters to punish (Sigmund et al., 2010, 2011). However, as we show here, this does not necessarily imply that taxes were wasted. When sanctioning institutions are publicly visible, they act as a costly signal. Their mere existence may be the reason why there were no exploiters to punish in the first place (and conversely, this deterrent effect of sanctioning institutions may be the major incentive to make the institutions publicly visible).

In the following, we present a model that makes this signaling effect of sanctioning institutions explicit, based on the public goods game (PGG) by Sigmund et al. (2010). In our model, which is formally introduced in Section 2, individuals can condition their behavior in the PGG on whether or not a punishment institution has been established. This extended model gives rise to new opportunistic behaviors: individuals may decide to cooperate in the PGG only if they would be punished otherwise. We show in Section 3 that the possibility of opportunism facilitates the evolution of pool punishment systems even when participation in the PGG is mandatory, and even when tax evaders are not punished, i.e. in the absence of second-order punishment. This implies that in contrast to previous work (Sigmund et al., 2010; Traulsen et al., 2012), second-order punishment is not required for the stability of the punishment institution if the institution׳s existence is known before the public goods game takes place. Thus, our model considerably extends the range of application of pool punishment institutions, as compared to the original model by Sigmund et al. (2010). Without second-order punishment, however, tax evasion remains a persistent phenomenon. Evolution settles at a mixed equilibrium in which a majority of individuals cooperate in the PGG, whereas only a minority pays taxes for the punishment institution.

In Section 4 we discuss the robustness of our results by considering four different model extensions. With the first two extensions, diminishing taxes and second-order punishment, we explore two different mechanisms that aim to reduce tax evasion. Only the second model extension will turn out to be an effective means to eradicate tax evasion completely. In the third model extension we explore the impact of incomplete information. When punishment institutions are not perfectly visible, opportunists may fail to adapt, which in turn reduces the institution׳s efficiency. Nevertheless, we will show that perfect information is not necessary for stable pool punishment to evolve. The last model extension then explores the impact of different punishment technologies. In Section 5, we discuss these findings and compare our results to previous literature on institution formation.

## 2 Baseline model

### 2.1 Setup of the public goods game

We consider a PGG between *n* players and with three stages. In the first stage, all individuals decide whether to participate in the game or not. Non-participants, the so-called loners, do not have to take any other decision and get a small, but secure payoff σ>0, which is independent of the decisions of the other players (Hauert et al., 2002a,b). In the second stage, participants choose whether or not to pay taxes, i.e., whether to pay an amount γ>0 for the punishment institution. In the last stage of the game, participants are informed on the establishment of the punishment institution and they can choose whether to contribute an amount c>0 to the public good. Contributions are multiplied by a factor r>1, and equally shared among all *other* participants (i.e., contributions can be considered as purely altruistic, as individuals do not benefit from their own contributions. This variant of the public goods game is sometimes referred to as “mutual aid game”, see e.g. De Silva et al., 2010; Zhang et al., 2013; Sasaki, 2013). Moreover, if at least one player paid taxes for the punishment institution, then all non-contributors have to pay a fine *β*. Later, we will relax this assumption and consider situations where more tax payers lead to a more severe punishment. In order for the model to be interesting, we suppose that β>c (such that punishment fines act as a deterrent) and that σ<rc−c−γ (such that a society where everyone pays taxes and contributes to the public good is superior to a society where subjects simply abstain from collective action).

As the main difference to the pool punishment model of Sigmund et al. (2010), we assume that participants know whether a punishment institution has been established *before* they are asked to contribute to the public good. Thus, in addition to the previously considered set of strategies, consisting of loners *L* (who abstain from the PGG), defectors *D* (who neither pay taxes nor contribute to the public good), cooperators *C* (who contribute to the public good, but do not pay taxes), and pool punishers *P* (who both pay taxes and contribute to the common pool), we also allow for opportunists *O*. Opportunists do not pay taxes, and they contribute to the public good only if there is a punishment institution.

Note that there are two additional possible types: anti-opportunists *A* (who do not pay taxes and only contribute to the public pool if no punishment institution has been established) and self-defeating players *S* (who pay taxes but fail to contribute to the public good). These two behaviors are somewhat paradoxical, and one can easily show that evolutionary processes lead to their extinction if selection pressure is sufficiently strong. For the sake of simplicity, we will thus neglect these two strategies in the following (our main results remain essentially unchanged if we considered the complete strategy space instead – only if selection is weak, all available strategies would be present).

### 2.2 Population setup and evolutionary dynamics

To model the evolutionary dynamics of strategies over time, we suppose that the above PGG is played in a population of finite size *N*. Let *N*_*L*_, *N*_*D*_, *N*_*C*_, *N*_*P*_ and *N*_*O*_ denote respectively the number of loners, defectors, cooperators, pool punishers and opportunists in the population, such that $N_{L}+N_{D}+N_{C}+N_{P}+N_{O}=N$. We assume that groups for the PGG are formed randomly, by drawing *n* individuals from the population without replacement. In that case, the expected payoff *π*_*i*_ for each strategy i∈{L,C,D,P,O} can be computed by averaging over all possible group compositions obtained by multivariate hypergeometric sampling (see Appendix). The resulting payoffs become

(1) $$\pi_{L}=\sigma \pi_{D}=\frac{\left(\begin{array}{c} N_{L}\\ n-1 \end{array}\right)}{\left(\begin{array}{c} N-1\\ n-1 \end{array}\right)}\sigma +(1-\frac{\left(\begin{array}{c} N_{L}\\ n-1 \end{array}\right)}{\left(\begin{array}{c} N-1\\ n-1 \end{array}\right)})(\mathrm{rc}\frac{N-N_{L}-N_{D}}{N-N_{L}-1}-\beta )-\frac{\left(\begin{array}{c} N-N_{P}-1\\ n-1 \end{array})-(\begin{array}{c} N_{L}\\ n-1 \end{array}\right)}{\left(\begin{array}{c} N-1\\ n-1 \end{array}\right)}(\mathrm{rc}\frac{N_{O}}{N-N_{L}-N_{P}-1}-\beta )\pi_{C}=\frac{\left(\begin{array}{c} N_{L}\\ n-1 \end{array}\right)}{\left(\begin{array}{c} N-1\\ n-1 \end{array}\right)}\sigma +(1-\frac{\left(\begin{array}{c} N_{L}\\ n-1 \end{array}\right)}{\left(\begin{array}{c} N-1\\ n-1 \end{array}\right)})(\mathrm{rc}\frac{N-N_{L}-N_{D}-1}{N-N_{L}-1}-c)-\frac{\left(\begin{array}{c} N-N_{P}-1\\ n-1 \end{array})-(\begin{array}{c} N_{L}\\ n-1 \end{array}\right)}{\left(\begin{array}{c} N-1\\ n-1 \end{array}\right)}\mathrm{rc}\frac{N_{O}}{N-N_{L}-N_{P}-1}\pi_{P}=\frac{\left(\begin{array}{c} N_{L}\\ n-1 \end{array}\right)}{\left(\begin{array}{c} N-1\\ n-1 \end{array}\right)}\sigma +(1-\frac{\left(\begin{array}{c} N_{L}\\ n-1 \end{array}\right)}{\left(\begin{array}{c} N-1\\ n-1 \end{array}\right)})(\mathrm{rc}\frac{N-N_{L}-N_{D}-1}{N-N_{L}-1}-c-\gamma )\pi_{O}=\frac{\left(\begin{array}{c} N_{L}\\ n-1 \end{array}\right)}{\left(\begin{array}{c} N-1\\ n-1 \end{array}\right)}\sigma +(1-\frac{\left(\begin{array}{c} N_{L}\\ n-1 \end{array}\right)}{\left(\begin{array}{c} N-1\\ n-1 \end{array}\right)})(\mathrm{rc}\frac{N-N_{L}-N_{D}-1}{N-N_{L}-1}-c)-\frac{\left(\begin{array}{c} N-N_{P}-1\\ n-1 \end{array})-(\begin{array}{c} N_{L}\\ n-1 \end{array}\right)}{\left(\begin{array}{c} N-1\\ n-1 \end{array}\right)}(\mathrm{rc}\frac{N_{O}-1}{N-N_{L}-N_{P}-1}-c)$$

To interpret these payoffs, let us take the payoff of a defector *π*_*D*_ as an example. The first expression on the right-hand side corresponds to the case of a group where all *n*−1 co-players of the defector are loners, such that no public goods game takes place and the defector obtains the loner׳s payoff *σ* automatically. The second term then gives the expected payoff when the defector plays the PGG, assuming that there is at least one pool punisher among the co-players. In that case, the defector obtains an expected benefit $\mathrm{rc}(N-N_{L}-N_{D})/(N-N_{L}-1)$, but in return has to pay a fine *β* due to the presence of the punishment institution. The last expression on the right-hand side is a correction term that accounts for the fact that there does not need to be a pool punisher among the defector׳s co-players. In that case, the defector saves the punishment fine *β*, but his expected benefit from the PGG is reduced by ${\mathrm{rcN}}_{O}/(N-N_{L}-N_{P}-1)$ due to the presence of opportunists who do not contribute in this case. Similar interpretations can be given for the other payoff values.

The frequency of each strategy in the population may change over time, depending on the strategy׳s relative success. To model such an evolutionary dynamics where successful strategies spread within the population, we employ a pairwise comparison process (Blume, 1993; Szabó and Tőke, 1998; Traulsen et al., 2006). That is, we assume that in each time step two players with strategies *i* and *j* are picked at random from the population. Depending on the payoffs of the two players, the *i*-player adopts strategy *j* with probability:

(2) $$p_{\mathrm{ij}}=\frac{1}{1+\exp[-s(\pi_{j}-\pi_{i})]}.$$

The parameter s≥0 is called the strength of selection. In the limit of weak selection, s→0, the probability *p*_*ij*_ approaches 1/2 independent of the payoffs, such that imitation occurs essentially at random. In the limit of strong selection, s→∞, on the other hand, players only imitate their co-players if the co-player yielded a higher payoff.

In addition to these social learning events, we allow for random strategy exploration (corresponding to mutations in genetic models). That is, we assume that in each time step, one player may switch to another strategy with probability μ>0 (with all other strategies having equal chance to be chosen). Overall, these assumptions imply that the change of strategies over time follows an ergodic stochastic process. When mutations are sufficiently rare, this process can be approximated by considering the transitions between homogeneous populations only (Fudenberg and Imhof, 2006; Wu et al., 2012).

## 3 Results

### 3.1 Static analysis

To obtain an intuitive understanding of the dynamics of the model, let us start by analyzing the evolutionary stable states (ESS) of the system; that is, we calculate the stable strategy mixtures such that no absent mutant strategy can invade (Maynard Smith and Price, 1973; Hofbauer and Sigmund, 1998).

**Fig. 1:**
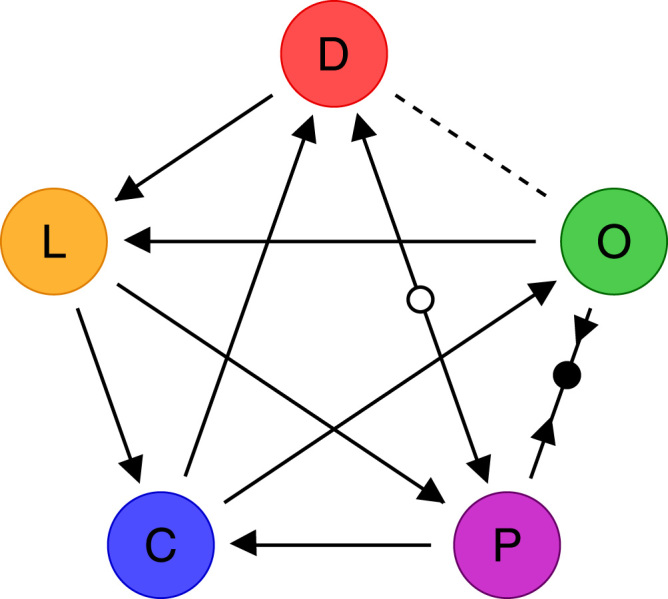
Schematic illustration of the possible transitions between homogeneous populations. Each colored circle corresponds to a homogeneous state where all members of the population play the same strategy. Black circles depict mixed equilibria between two pure strategies, and these circles are filled if the equilibrium is evolutionarily stable. Arrows describe the evolutionary transitions of the evolutionary process in the limit of rare mutations and strong selection (Fudenberg et al., 2006), with dashed lines denoting neutral drift. The graph indicates that there is no pure ESS, but there is a mixed ESS in which opportunists coexist with pool punishers. This ESS can be reached from any other initial population by following an appropriate transition path. For this illustration, we have assumed that β>γ+c, such that a population of pool punishers is stable against invasion by defectors.

As our first step, we note that there is no ESS in pure strategies. Among loners, cooperators, and defectors there is an evolutionary rock-scissors-paper cycle, with defectors invading cooperators, loners invading defectors, and cooperators invading loners (Hauert et al., 2002a, 2002b; Semmann et al., 2003), see also Fig. 1. A similar cycle characterizes the dynamics among loners, cooperators, opportunists, as the latter always act as defectors in the absence of the threat of punishment. As a consequence, if only defectors and opportunists are present in the population, the dynamics is governed by neutral drift. Between defectors and pool punishers there is a bistable competition, with none of the two strategies being able to invade the other given sufficiently large *N* and that β>c+γ. Nevertheless, a monomorphic population of pool punishers can always be invaded by cooperators, as cooperators save the costs for the punishment authority (i.e., cooperators are secondorder free riders, Sigmund et al., 2010). Thus, no pure ESS exists.

Interestingly, however, there is an evolutionarily stable mixture of pool punishers and opportunists. Indeed, a single opportunist in a population of pool punishers acts like a cooperator, and thus can invade as a second-order free rider. Conversely, a single pool punisher in a population of opportunists is able to induce full cooperation within his group, whereas groups of opportunists only end up with a payoff of zero. To calculate the equilibrium point between opportunists and pool punishers, we set $N_{L}=N_{C}=N_{D}=0$ in Eq. (1). Then the condition $\pi_{P}=\pi_{O}$ leads to

(3) $$\mathrm{rc}-c-\gamma =\mathrm{rc}-c-\frac{\left(\begin{array}{c} N_{O}-1\\ n-1 \end{array}\right)}{\left(\begin{array}{c} N-1\\ n-1 \end{array}\right)}(\mathrm{rc}-c)$$

For sufficiently large population sizes *N*, we can make use of the approximation

$$\frac{\left(\begin{array}{c} N_{O}-1\\ n-1 \end{array}\right)}{\left(\begin{array}{c} N-1\\ n-1 \end{array}\right)}\approx x_{O}^{n-1}$$

where $x_{O}=N_{O}/N$ denotes the fraction of opportunists in the population. Thus, for large populations the equilibrium fraction of opportunists according to condition (3) is approximately given by

(4) x^O=γrc−cn−1.

As 0<rc−c−γ, this value of x^O is always in the unit interval. The average payoff in this equilibrium is rc−c−γ (which coincides with the payoff of a homogeneous population of pool punishers). The payoff of the opportunists is reduced to this quantity as they often end up in groups where no one is willing to punish and thus no one cooperates.

One can easily show that this mixture of opportunists and pool punishers cannot be invaded by any of the three absent strategies *L*, *C*, or *D*: Loners cannot invade since σ<rc−c−γ. Cooperators and defectors cannot invade because Eq. (1) implies that both strategies are weakly dominated by the opportunistic strategy (i.e. $\pi_{C}\leq \pi_{O}$ and $\pi_{D}\leq \pi_{O}$ for all possible population mixtures; for 0<x^O<1 both inequalities are strict in sufficiently large populations). With a similar argument, one can also show that the game has no other ESS. Moreover, this unique ESS can be reached from any given initial population (see Fig. 1).

Thus our static analysis predicts that for sufficiently strong selection, evolutionary dynamics will lead to a stable coexistence of pool punishers and opportunists. According to Eq. (4), the equilibrium fraction of opportunists increases with the tax amount *γ* and with group size *n*, and it decreases with the net benefit of the public good, *rc*-*c*.

### 3.2 Numerical simulations

**Fig. 2:**
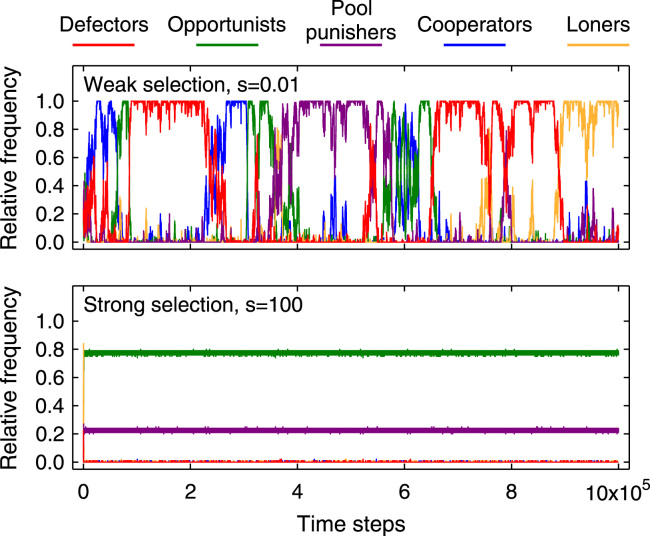
Evolutionary dynamics for two different selection regimes. Each graph shows a representative simulation run for the case of weak selection (upper graph) and strong selection (lower graph), respectively. For both runs, mutations are sufficiently rare such that mutants typically take over the whole population or go extinct before the next mutation arises. While the weak selection regime exhibits a strong oscillatory behavior, the strong selection regime leads to a stable coexistence of opportunists and pool punishers. Parameters: *N*=100, *n*=5, *c*=1, *r*=3, *γ*=0.7, *β*=1.5, *σ*=1 and *μ*=0.001.

In order to confirm these analytical predictions and to explore other intensities of selection, we have run individual-based simulations of the evolutionary process as described in Section 2.2. Assuming rare mutations, we find two different dynamical regimes (see Fig. 2). When selection is weak (*s*=0.01), and payoffs only play a subordinate role for evolutionary success, no strategy is able to get the upper hand. Instead, there are endless cycles that lead from one homogeneous population to another homogeneous population. In this regime, loners are most abundant, as a population of loners is most robust against invasion by other strategies: leaving a population of loners requires not only a mutant that switches to cooperation or pool punishment, but also this single mutant is the only participant of the game and acts as a loner. Only if the single mutant successfully reproduces (which happens with probability 1/2), it can take over the population. For all other homogeneous populations, one appropriate mutant is immediately advantageous and can lead the population away from the corresponding state (for example, one single defector is advantageous in a population of cooperators).

For strong selection (*s*=100) the qualitative behavior is clearly different: the population almost immediately converges to a stable mixture of opportunists and pool punishers. The position of this stable mixture is accurately predicted by Eq. (4): for the parameters of the simulation the stable fraction of opportunists becomes x^O=0.7/24≈0.77. Thus, only a minority of players pays taxes in this equilibrium. However, this is still sufficient to reach a substantial amount of cooperation in the PGG: the probability that all players of a randomly formed group contribute to the public good is given by 1−x^On≈0.73, and the payoff in this equilibrium is rc−c−γ=1.3. Thus, publicly visible sanctioning institutions are able to induce a satisfactory level of cooperation, despite a substantial amount of second-order free riders in the population.

**Fig. 3:**
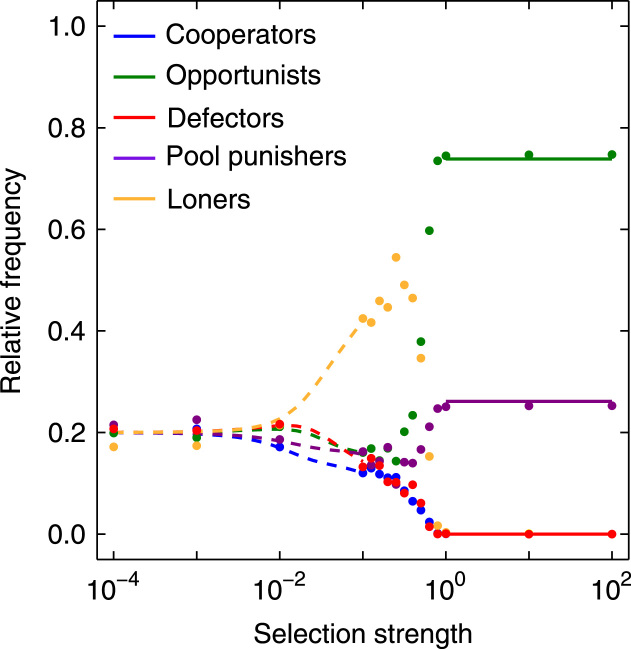
Long-run abundance of strategies for varying selection strength. Each point shows the outcome of an individual-based simulation. Dashed lines represent the analytical prediction based on the rare-mutation limit (Fudenberg and Imhof, 2006; Wu et al., 2012) which is only valid for weak selection in our case (as it presumes that populations do not settle at mixed states). Solid lines depict the prediction for strong selection based on the ESS of the system (Section 3.1). The rare-mutation limit gives a reasonable approximation of the dynamics for s<0.1, whereas for s>1, the system can be described by the ESS. Thus, for sufficiently strong selection only pool punishers and opportunists are present in the population; loners, defectors and cooperators are driven to extinction. To get a more detailed picture of the behavior when 0.1<s<1, we have run additional simulations for this interval. Parameters are the same as in Fig. 2, and simulations were run for 10^9^ time steps.

To find the critical selection intensity that separates the weak selection regime from the strong selection regime, we have run extensive simulations for a broad range of selection strengths. As depicted by Fig. 3, the weak selection regime can be found for selection strengths s≤0.1. For this parameter range, the long-run abundance of strategies can be reasonably predicted by the rare-mutation limit, which approximates the dynamics by only considering the Markov chain for transitions among the five homogeneous populations (Fudenberg and Imhof, 2006; Wu et al., 2012). As the selection strength exceeds s≈1, however, the long-run abundance of strategies is determined by the position of the ESS. In this regime, payoff differences have a sufficient impact on imitation for opportunists and pool punishers to be stable against invasion of other types.

### 3.3 Interpretation

For a better understanding of the importance of the signaling effect of institutions on the emergence of pool punishment, let us compare these findings with the results when such an effect is absent (Sigmund et al., 2010). If the signaling effect is absent, opportunism is not a possible behavior any longer, and the feasible strategy set consists of cooperators, defectors, loners and pool punishers only.

**Fig. 4:**
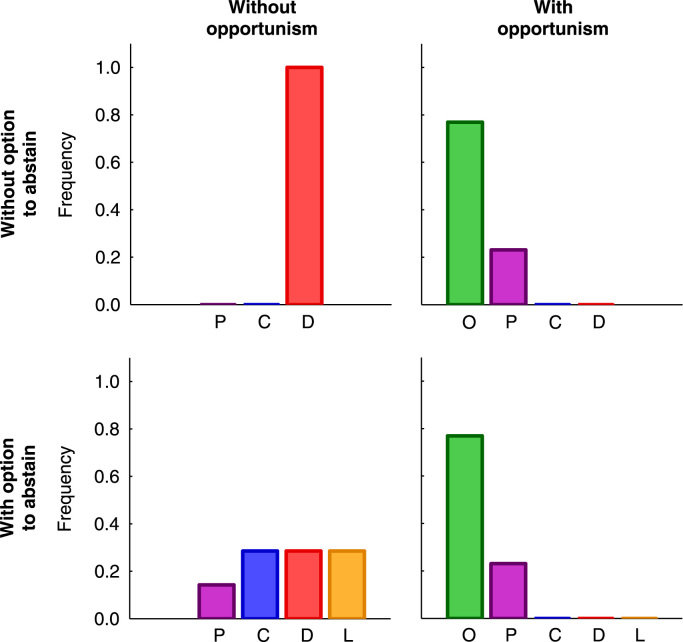
Effect of the option to abstain and the possibility of opportunism on the evolution of pool punishment. Each graph shows the distribution of strategies in the analytical limit of small mutation rates and strong selection. When opportunism is possible (i.e. when institutions are publicly visible), then the option to abstain is not necessary to establish a positive level of pool punishment, resulting in high levels of cooperation. In the case without opportunism, the result is parameter independent as long as the inequalities 0<σ<rc−c−γ and β>γ+c are satisfied (Sigmund et al., 2010, 2011). For the case with opportunism, we have used the previous parameters (Fig. 2) to depict the evolutionarily stable mixture of opportunists and pool punishers.

In Fig. 4, we show the frequency of strategies in the strong selection limit, depending on whether or not opportunism is feasible, and depending on whether or not abstaining from the public good is feasible. If neither option is feasible, evolution leads to a homogeneous population of free riders with payoff zero. The inclusion of an outside option, by adding loners to the population, can partly resolve the problem: in the new steady state, the strategies *L*, *D*, *C*, and *P* are played in proportions 2:2:2:1 (Sigmund et al., 2010, 2011). For the parameters used in the previous simulations, this implies that the long-run average payoff is approximately given by (2σ+3rc−3c−γ)/7≈1.07, which is clearly better than the defector׳s payoff of zero. The outside option thus improves the situation, because it provides an escape from fully selfish populations, without establishing stable cooperation, however.

In contrast, when punishment institutions are implemented such that they are visible for all community members, then a substantial level of cooperation can be achieved even if there is no outside option that helps to escape from defectors. Instead, a homogeneous population of defectors can be subverted by opportunists, initially through neutral drift (see also Fig. 1) – the option to abstain from the public goods interaction is no longer necessary as an escape hatch out of mutual defection. Once opportunism is common, such that a substantial fraction of the population can be swayed by additional external incentives, pool punishers can invade as they are able to transform non-cooperative co-players into fully cooperative co-players. Thus, the signaling effect of sanctioning institutions helps to overcome both problems, the emergence of cooperation in a population of defectors, and the continuous maintenance of a substantial level of cooperation over time.

## 4 Model extensions

### 4.1 Diminishing taxes

In the previous analysis, paying taxes for a punishment institution shares the characteristics of a volunteer׳s dilemma: it takes one volunteer who pays the taxes for a central institution that is beneficial for the whole group (Diekmann, 1985; Archetti and Scheuring, 2012; Raihani and Bshary, 2011; Przepiorka and Diekmann, 2013). However, this model has the maybe somewhat unrealistic feature that the total costs of the punishment institution increase linearly with the number of tax payers; in a group with *k* tax payers, the costs of the punishment institution are *γk*. Seen from this angle, it is of little surprise that the model predicts a considerable fraction of tax evaders: it would be inefficient if everyone paid taxes.

Instead, one may consider a model in which the total costs of a punishment institution are fixed to *γ*, and these costs are shared among all tax-payers (Weesie and Franzen, 1998). Under this assumption, individual taxes decrease with the number of tax payers. In a group with *k* tax payers, each tax payer only has to pay *γ*/*k*. Leaving everything else unchanged, one may speculate that such diminishing taxes are able to raise the fraction of pool punishers in the population, and that they suppress tax evasion.

It is relatively simple to incorporate diminishing taxes into our model (for peer punishment systems in infinitely large populations, this has been done by Dercole et al., 2013). Only the expected payoff of a pool punisher needs to be replaced by

(5) $$\pi_{P}=\frac{\left(\begin{array}{c} N_{L}\\ n-1 \end{array}\right)}{\left(\begin{array}{c} N-1\\ n-1 \end{array}\right)}\sigma +(1-\frac{\left(\begin{array}{c} N_{L}\\ n-1 \end{array}\right)}{\left(\begin{array}{c} N-1\\ n-1 \end{array}\right)})(\mathrm{rc}\frac{N-N_{L}-N_{D}-1}{N-N_{L}-1}-c)-\frac{\gamma }{{\mathrm{nN}}_{P}/N}(1-\frac{\left(\begin{array}{c} N-N_{P}\\ n \end{array}\right)}{\left(\begin{array}{c} N\\ n \end{array}\right)})+\frac{\left(\begin{array}{c} N_{L}\\ n-1 \end{array}\right)}{\left(\begin{array}{c} N-1\\ n-1 \end{array}\right)}\gamma .$$

One can interpret this modified payoff $\pi_{P}$ as follows: if *N*_*P*_ is large, then the expected number of punishers in the group is roughly $n\cdot N_{P}/N$, and thus the expected individual tax is approximately $\gamma /({\mathrm{nN}}_{P}/N)$. However, two corrections are necessary; one that accounts for the fact that the group contains at least one punisher, and the other for the fact that no tax needs to be paid when all other players are loners.

To calculate the ESS of the modified system with diminishing taxes, we proceed as in Section 3.1. For $N_{L}=N_{D}=N_{C}=0$, the condition $\pi_{P}=\pi_{O}$ is equivalent to

(6) $$\mathrm{rc}-c-\frac{\gamma }{{\mathrm{nN}}_{P}/N}(1-\frac{\left(\begin{array}{c} N-N_{P}\\ n \end{array}\right)}{\left(\begin{array}{c} N\\ n \end{array}\right)})=\mathrm{rc}-c-\frac{\left(\begin{array}{c} N_{O}-1\\ n-1 \end{array}\right)}{\left(\begin{array}{c} N-1\\ n-1 \end{array}\right)}(\mathrm{rc}-c).$$

In the limit of large population sizes, this equation becomes

(7) $$\mathrm{rc}-c-\frac{\gamma }{n(1-x_{O})}(1-x_{O}^{n})=(\mathrm{rc}-c)\cdot (1-x_{O}^{n-1}).$$

This can be reduced to the equation $f(x_{O})=0$, with

(8) $$f(x_{O})=(\mathrm{rc}-c)x_{O}^{n-1}-\frac{\gamma }{n}(x_{O}^{n-1}+x_{O}^{n-2}+\cdots +x_{O}+1).$$

As this is a polynomial of degree *n*−1, there is no analytical expression for the equilibrium fraction of opportunists x^O. However, for the parameter values used before (see Fig. 2), one can solve $f(x_{O})=0$ numerically, yielding the unique real solution within the unit interval x^O≈0.65 (as compared to x^O=0.77 in the equilibrium without diminishing taxes). The resulting average payoff in the population is π^≈1.65. Thus, diminishing taxes indeed have the potential to reduce tax evasion, and to increase average payoffs. Overall, however, tax evasion remains a persistent phenomenon (*x*_*O*_=0 is never an equilibrium as f(0)<0 for all parameter values).

### 4.2 Second-order punishment

As another mechanism that may reduce tax evasion, let us briefly consider second-order punishment (in which case not only non-contributors are punished, but additionally also tax-evaders). Assuming that the penalty for each offense is *β* (such that non-contributing tax evaders pay a fine 2*β*), this leads to the following modification of the payoffs in Eq. (1):

(9) $$\pi_{D}=\frac{\left(\begin{array}{c} N_{L}\\ n-1 \end{array}\right)}{\left(\begin{array}{c} N-1\\ n-1 \end{array}\right)}\sigma +(1-\frac{\left(\begin{array}{c} N_{L}\\ n-1 \end{array}\right)}{\left(\begin{array}{c} N-1\\ n-1 \end{array}\right)})(\mathrm{rc}\frac{N-N_{L}-N_{D}}{N-N_{L}-1}-2\beta )-\frac{\left(\begin{array}{c} N-N_{P}-1\\ n-1 \end{array})-(\begin{array}{c} N_{L}\\ n-1 \end{array}\right)}{\left(\begin{array}{c} N-1\\ n-1 \end{array}\right)}(\mathrm{rc}\frac{N_{O}}{N-N_{L}-N_{P}-1}-2\beta )\pi_{C}=\frac{\left(\begin{array}{c} N_{L}\\ n-1 \end{array}\right)}{\left(\begin{array}{c} N-1\\ n-1 \end{array}\right)}\sigma +(1-\frac{\left(\begin{array}{c} N_{L}\\ n-1 \end{array}\right)}{\left(\begin{array}{c} N-1\\ n-1 \end{array}\right)})(\mathrm{rc}\frac{N-N_{L}-N_{D}-1}{N-N_{L}-1}-c-\beta )-\frac{\left(\begin{array}{c} N-N_{P}-1\\ n-1 \end{array})-(\begin{array}{c} N_{L}\\ n-1 \end{array}\right)}{\left(\begin{array}{c} N-1\\ n-1 \end{array}\right)}(\mathrm{rc}\frac{N_{O}}{N-N_{L}-N_{P}-1}-\beta )\pi_{P}=\frac{\left(\begin{array}{c} N_{L}\\ n-1 \end{array}\right)}{\left(\begin{array}{c} N-1\\ n-1 \end{array}\right)}\sigma +(1-\frac{\left(\begin{array}{c} N_{L}\\ n-1 \end{array}\right)}{\left(\begin{array}{c} N-1\\ n-1 \end{array}\right)})(\mathrm{rc}\frac{N-N_{L}-N_{D}-1}{N-N_{L}-1}-c-\gamma )\pi_{O}=\frac{\left(\begin{array}{c} N_{L}\\ n-1 \end{array}\right)}{\left(\begin{array}{c} N-1\\ n-1 \end{array}\right)}\sigma +(1-\frac{\left(\begin{array}{c} N_{L}\\ n-1 \end{array}\right)}{\left(\begin{array}{c} N-1\\ n-1 \end{array}\right)})(\mathrm{rc}\frac{N-N_{L}-N_{D}-1}{N-N_{L}-1}-c-\beta )-\frac{\left(\begin{array}{c} N-N_{P}-1\\ n-1 \end{array})-(\begin{array}{c} N_{L}\\ n-1 \end{array}\right)}{\left(\begin{array}{c} N-1\\ n-1 \end{array}\right)}(\mathrm{rc}\frac{N_{O}-1}{N-N_{L}-N_{P}-1}-c-\beta ),$$

with the payoff for loners being unchanged, $\pi_{L}=\sigma$. Provided that β>γ, it is straightforward to show that a homogeneous population of pool punishers now is evolutionarily stable: in such a population, the payoff of the residents is rc−c−γ, whereas the payoff of a single mutant is $\pi_{D}=\mathrm{rc}-2\beta$ (if the mutant is a defector), $\pi_{C}=\pi_{O}=\mathrm{rc}-c-\beta$ (if the mutant is a cooperator or an opportunist), or $\pi_{L}=\sigma$ (if the mutant is a loner); thus, our assumptions β>c and σ<rc−c−γ guarantee that all mutant payoffs are below the payoff of the resident.

As in Sigmund et al. (2010), we arrive at the conclusion that second-order punishment leads to the fixation of pool punishment. The mean payoff in such a population, however, is π^=rc−c−γ, which is exactly the same mean payoff as in the model without second-order punishment. Thus, in contrast to the case of diminishing taxes, second-order punishment is effective to eradicate tax evasion, but it does not lead to any improvement of the average payoffs. If the two mechanisms can be combined, however, the mean payoff becomes π^=rc−c−γ/n (or, with our parameter values, π^=1.86), which converges to the social optimum *rc*-*c* for large groups.

### 4.3 Incomplete information

The previous findings can be considered as a proof of principle, showing that public punishment institutions can facilitate the evolution of pool punishment. To this end, we have assumed that once a punishment institution is established, its existence immediately becomes common knowledge, and all individuals have the chance to adapt their behaviors correspondingly. Let us now investigate the consequences of incomplete information: instead of assuming that all players are informed about the existence of a punishment institution, we now assume that only a fraction *λ* of individuals knows about the institution׳s existence (with 0≤λ≤1). Moreover, let us suppose that the players׳ information does not depend on their strategy, and that opportunists cooperate if they know that a punishment institution has been established and defect otherwise. In that case, the expected payoffs need to be modified as follows:

(10) $$\pi_{D}=\frac{\left(\begin{array}{c} N_{L}\\ n-1 \end{array}\right)}{\left(\begin{array}{c} N-1n-1 \end{array}\right)}\sigma +(1-\frac{\left(\begin{array}{c} N_{L}\\ n-1 \end{array}\right)}{\left(\begin{array}{c} N-1\\ n-1 \end{array}\right)})(\mathrm{rc}\frac{N_{C}+N_{P}+\lambda N_{O}}{N-N_{L}-1}-\beta )-\frac{\left(\begin{array}{c} N-N_{P}-1\\ n-1 \end{array})-(\begin{array}{c} N_{L}\\ n-1 \end{array}\right)}{\left(\begin{array}{c} N-1\\ n-1 \end{array}\right)}(\mathrm{rc}\frac{\lambda N_{O}}{N-N_{L}-N_{P}-1}-\beta )\pi_{C}=\frac{\left(\begin{array}{c} N_{L}\\ n-1 \end{array}\right)}{\left(\begin{array}{c} N-1\\ n-1 \end{array}\right)}\sigma +(1-\frac{\left(\begin{array}{c} N_{L}\\ n-1 \end{array}\right)}{\left(\begin{array}{c} N-1\\ n-1 \end{array}\right)})(\mathrm{rc}\frac{N_{C}+N_{P}+\lambda N_{O}-1}{N-N_{L}-1}-c)-\frac{\left(\begin{array}{c} N-N_{P}-1\\ n-1 \end{array})-(\begin{array}{c} N_{L}\\ n-1 \end{array}\right)}{\left(\begin{array}{c} N-1\\ n-1 \end{array}\right)}\mathrm{rc}\frac{\lambda N_{O}}{N-N_{L}-N_{P}-1}\pi_{P}=\frac{\left(\begin{array}{c} N_{L}\\ n-1 \end{array}\right)}{\left(\begin{array}{c} N-1\\ n-1 \end{array}\right)}\sigma +(1-\frac{\left(\begin{array}{c} N_{L}\\ n-1 \end{array}\right)}{\left(\begin{array}{c} N-1\\ n-1 \end{array}\right)})(\mathrm{rc}\frac{N_{C}+N_{P}+\lambda N_{O}-1}{N-N_{L}-1}-c-\gamma )\pi_{O}=\frac{\left(\begin{array}{c} N_{L}\\ n-1 \end{array}\right)}{\left(\begin{array}{c} N-1\\ n-1 \end{array}\right)}\sigma +(1-\frac{\left(\begin{array}{c} N_{L}\\ n-1 \end{array}\right)}{\left(\begin{array}{c} N-1\\ n-1 \end{array}\right)})(\mathrm{rc}\frac{N_{C}+N_{P}+\lambda (N_{O}-1)}{N-N_{L}-1}-\lambda c-(1-\lambda )\beta )-\frac{\left(\begin{array}{c} N-N_{P}-1\\ n-1 \end{array})-(\begin{array}{c} N_{L}\\ n-1 \end{array}\right)}{\left(\begin{array}{c} N-1\\ n-1 \end{array}\right)}(\mathrm{rc}\frac{\lambda (N_{O}-1)}{N-N_{L}-N_{P}-1}-\lambda c-(1-\lambda )\beta ),$$

with the payoff for loners being unchanged, $\pi_{L}=\sigma$. Using the large population approximation as before, we can calculate the mixed equilibrium between opportunists and pool punishers. We find that this equilibrium only exists if the information level *λ* is above a critical threshold, $\lambda_{1}=(c+\gamma )/\mathrm{rc}$, in which case the equilibrium fraction of opportunists is given by

(11) x^O=γ−(1−λ)(β−c)λ(rc−c)−(1−λ)βn−1.

**Fig. 5:**
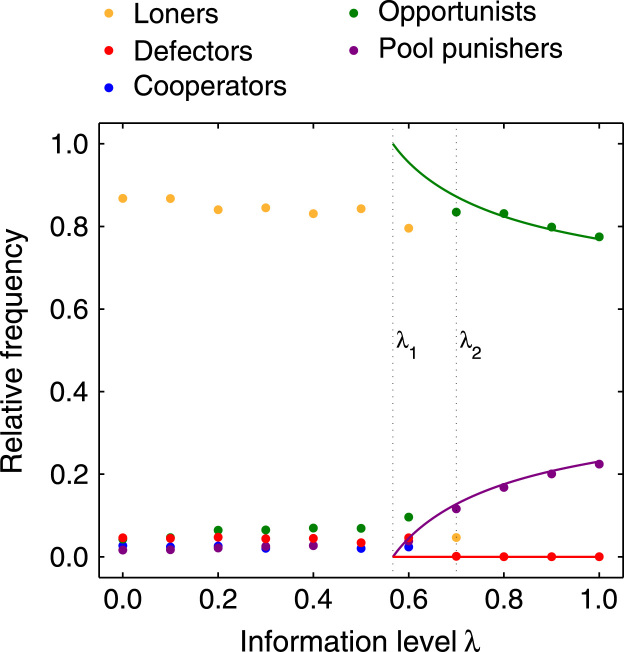
Evolutionary dynamics under incomplete information. Each point shows the abundance of the respective strategy in simulations for various information levels *λ*. Public sanctioning institutions only promote the evolution of pool punishment if players have a sufficient knowledge about the institution׳s existence. The equilibrium between pool punishers and opportunists (depicted by lines) exists if *λ* exceeds the threshold $\lambda_{1}=(c+\gamma )/\mathrm{rc}$, and it is stable if $\lambda >\lambda_{2}$ (where the equilibrium payoff exceeds the loner׳s payoff). Once the equilibrium is stable, it accurately describes the outcome of the simulation (parameters: *N*=100, *n*=5, *c*=1, *r*=3, *γ*=0.7, *β*=1.5, *σ*=0.5 and *μ*=0.001).

As expected, this fraction coincides with expression (4) in the case of full information, *λ*=1. The equilibrium is stable if equilibrium payoffs exceed the payoffs of loners and cooperators (which will always be the case if *λ* approaches 1); the corresponding threshold *λ*_2_ can be obtained numerically. Fig. 5 gives an illustration of these findings: public institutions do only promote pool punishment if they are *sufficiently* public. For the parameters shown here, roughly three quarters of the population need to be informed about the institution׳s existence. Interestingly, however, the payoff formulas in (10) indicate that all players benefit from a high information level – as all payoffs are monotonically increasing in *λ*. Intuitively, tax payers want the institution to be publicly visible as this increases the cooperation rate within their group, whereas opportunists benefit from public information because it helps them to avoid punishment.

### 4.4 Institutions with a linear punishment technology

In the previous models we have modeled the institution׳s punishment technology as a volunteer׳s dilemma: a single tax payer is sufficient to set up an effective punishment institution. This allowed a minority of tax payers to invade a population of opportunists, by successfully swaying them to cooperate. However, especially in large groups it will typically take a larger number of tax payers to create a reliable punishment regime. Does this prohibit the emergence of public sanctioning institutions?

To investigate this question, we have explored sanctioning institutions with a linear punishment technology, as in Sigmund et al. (2010, 2011): if *k* is the number of tax payers within a group, then free riders are punished by an amount βk. This model reflects a situation in which tax payers contribute an amount γk into a punishment pool which is then used to detect and sanction free riders. When β<c, a single tax payer is no longer sufficient to deter opportunists; instead it takes a least k^=⌈c/β⌉ tax payers to create a sufficient threat (i.e., k^ is the lowest integer such that βk^≥c).

**Fig. 6:**
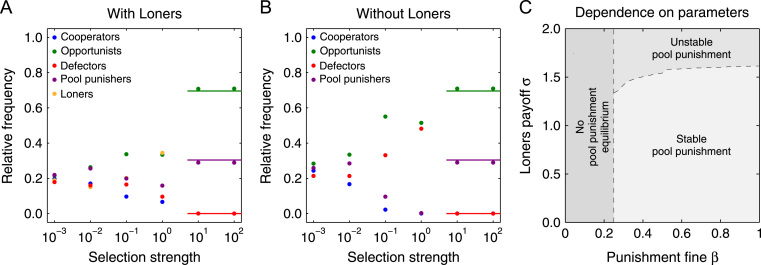
Evolution of stable institutions for a linear punishment regime. The panels (A) and (B) show the outcome of simulations (dots) together with the analytical prediction according to the stable equilibrium arising from Eq. (13) (lines). For the parameters used here, we recover the result that public institutions allow the emergence of stable pool punishment in the limit of strong selection. In general, such a result can be expected if the punishment technology is sufficiently efficient, and if abstaining from the public good is not too profitable, as depicted in panel (C) (parameters: *N*=100, *n*=10, *c*=1, *r*=3, *γ*=0.3, *β*=0.6, *σ*=0.5 and *μ*=0.001).

For Fig. 6 we have therefore run additional simulations for which we have assumed that opportunists only cooperate if they find themselves in a group in which the punishment institution is sufficiently powerful (i.e., if k≥k^). These simulations indicate that stable pool punishment institutions can still evolve under appropriate conditions. However, unlike in the previous models, the emergence and the stability of pool punishment depend on the exact parameters of the game. To see this, let us calculate the equilibrium conditions for a stable coexistence between pool punishers and opportunists in large populations. If all other strategies are absent, the payoffs of the two strategies are given by

(12) πO(xO)=∑nP=0k^−1(n−1nP)(1−xO)nPxOn−1−nP(rcn−1−β)nP+∑nP=k^n−1(n−1nP)(1−xO)nPxOn−1−nP(rc−c)πP(xO)=∑nP=0k^−2(n−1nP)(1−xO)nPxOn−1−nP(rcnPn−1−c−γ)+∑nP=k^−1n−1(n−1nP)(1−xO)nPxOn−1−nP(rc−c−γ)

The equilibrium fraction of opportunists can then be found by solving the implicit equation:

(13) $$g(x_{O})=\pi_{P}(x_{O})-\pi_{O}(x_{O})=0.$$

If the population consists of pool punishers only, then $g(x_{O}=0)=-\gamma <0$. On the other hand, if the population consists of opportunists only, then $g(x_{O}=1)=-c-\gamma <0$ (provided that k^>1). Thus, opportunists gain higher payoffs at both boundaries, and the existence of an interior equilibrium is no longer guaranteed.

Nevertheless, depending on the parameters of the game, a stable interior equilibrium may still exist, as in the case of Fig. 6. In that case, it follows as before that this equilibrium is globally stable if players have the option to abstain from the public good. Surprisingly, this equilibrium may even be attained if loners are removed from the strategy set (as depicted in Fig. 6B). In that case, the dynamics is bistable: populations either move towards a mixture of opportunists and defectors, or towards a mixture of opportunists and pool punishers. Which of these equilibria is eventually chosen depends on the respective basins of attraction, and therefore on the game parameters. In general, stable punishment institutions are most likely to evolve if the punishment technology is efficient (i.e., if *β*/*γ* is sufficiently high), and if abstaining from the public good is not too profitable (i.e., if *σ* is sufficiently low), as depicted in Fig. 6C.

In a similar way, one can also study sanctioning institutions where the punishment fine additionally depends on the number of free riders in a group (such that the funds in the punishment pool βk are divided by the number of free riders that are to be punished). Such a model would yield similar qualitative conclusions: again, it depends on the exact parameters of the game whether there is an equilibrium between pool punishers and opportunists. When there is such an equilibrium, it is either globally stable (if players have the option to abstain from the public good), or there is a bistable dynamics (if participation in the game is mandatory).

## 5 Discussion

During the last decades, there have been tremendous efforts to study how forms of punishment and policing can be used to uphold cooperation (for a summary of these efforts,we refer to Sigmund, 2007). Most previous models have considered peer punishment, assuming that cheaters are directly punished by their conspecifics (if the interaction occurred within a species), or by the fooled interaction partner (in mutualistic symbioses between species, as in Bshary and Grutter, 2005; Goto et al., 2010). In some cases, such as in humans (Fehr and Fischbacher, 2003) and in the cleaner fish mutualism (Raihani et al., 2010), it has even been observed that third parties may engage in costly punishment. As sanctions lead to a selective pressure against cheating, punishment can be considered as a theoretical solution for the problem of cooperation. This solution, however, opens up a series of new problems: What is the evolutionary advantage of engaging in punishment in the first place (Colman, 2006)? Why would unrelated third parties engage in punishment (Roos et al., 2014)? When is punishment used responsibly to punish cheaters only, and when is it misused for antisocial punishment or spiteful punishment (Hilbe and Traulsen, 2012)? These problems have led to the conclusion that peer punishment may be mainly used to establish dominance hierarchies, instead of maintaining mutual cooperation (Dreber et al., 2008).

Humans have found a rather unique way to circumvent some of these problems. Instead of relying on decentralized sanctioning mechanisms, many human societies have implemented norms and institutions to maintain cooperation (and conversely, some of the most pressing problems, such as the prevention of dangerous climate change, are still unsolved because of a lack of proper institutions on the global scale). The emergence and the stability of such institutions can be studied using evolutionary game theory, and the corresponding models, such as the model herein, share many features with models for the evolution of peer punishment (see also the models of Sigmund et al., 2010, 2011; Sasaki et al., 2012; Perc, 2012). However, there is an important difference between peer punishment and pool punishment: the costs of peer punishment are paid after cheating has taken place, whereas the costs of setting up a pool punishment institution need to be paid beforehand. Contributions to punishment institutions can therefore additionally act as a costly signal, which can be used to affect the behaviors of others. The signals in our model are not meant to confer information about individual quality, as in models of partner choice (e.g. Zahavi, 1975; Grafen, 1990; Han et al., 2013). Instead, these signals communicate that cheating will be unprofitable. Police stations, located in the middle of a town, do not only help to sanction rule-breakers; they prevent individuals from breaking the rules in the first place. Given this signaling effect of punishment institutions, it becomes in turn quite natural to locate police stations at publicly visible places.

Our model provides a link between evolutionary game theory and the literature on the economics of crime which interprets crime as a consequence of rational reasoning (e.g., Becker, 1968). According to this latter view, people only commit crime if the expected benefit exceeds the associated risks. This requires individuals to take into account all available signs that allow them to assess the prospects of cheating. In principle, a vast number of direct and indirect signs may be available for such an assessment. It has been argued that already a building with a broken window, which is left unrepaired, could act as such a signal (Wilson and Kelling, 1982; Keizer et al., 2008). The opportunists in our model employ a rudimentary form of this kind of reasoning – they are cooperative in a community that has established an effective sanctioning system, and they start cheating when such a system is absent. As our results suggest, such opportunistic behavior evolves quite naturally when sanctioning institutions act as a public signal. Interestingly, however, it is exactly the emergence of such opportunistic behavior that promotes the evolution of public punishment institutions and eventually stabilizes them.

As we have shown, neither the option to abstain nor second-order punishment is necessary for pool punishment to evolve if the signaling effect is present. This does not imply that second-order punishment is needless (in fact, in most societies tax evasion is illegal and not tolerated). Instead, we have shown that second-order punishment can be an effective means to reduce tax evasion, and to establish a fair division of the costs for a punishment institution (under the realistic assumption that individual taxes decrease with the number of tax payers). In many experiments, a fair division of institutional costs seems to be an important issue; subjects are unwilling to implement individually beneficial institutions if this would lead to unfair outcomes (e.g., Kosfeld et al., 2009). Effective institutions are therefore likely to require a mix of different mechanisms; they may employ second-order punishment to induce fair outcomes, and they may rely on the signaling effect to reduce the temptation to free ride.

Some explanations for the evolution of peer punishment are based on a similar signaling effect: subjects may punish others in order to establish a strict reputation, which may help them in future encounters (Sigmund et al., 2001; dos Santos et al., 2011; Hilbe and Traulsen, 2012; dos Santos et al., 2013). Reputation is an important driving force for cooperation (Leimar and Hammerstein, 2001; Milinski et al., 2002; Ohtsuki and Iwasa, 2004; Nowak and Sigmund, 2005; Bateson et al., 2006). With our model, we have aimed to extend the concept of reputation to institutions: central authorities like the police have a strict reputation by their very nature, which in turn may prevent community members from cheating. This may be especially relevant in anonymous communities: even if groups are too large to know the reputation of all community members, it seems fairly easy to communicate the existence of a central punishment institution. Thus, we speculate that this signaling effect may also be an important reason for the transition from decentralized peer punishment to centralized pool punishment.

We stress that signaling the willingness to punish others per se is not advantageous (Hilbe and Traulsen, 2012): in the case of peer punishment, only responsible punishment behavior targeted at free-riders can emerge based on opportunism, but not bullying or spite. A peer punishment system, however, has the disadvantage that it is based on threats that may be mere cheap talk. But in the case of centralized sanctions, where the institution has to be supported before the public goods interaction takes place, an honest signal can help cooperation to emerge and to prevail.

## Appendix A Derivation of payoffs

Herein, we derive the payoffs *π*_*i*_ for the four strategies i∈{D,C,P,O} according to Eq. (1). Suppose the focal player interacts with *n*_*L*_ loners, *n*_*D*_ defectors, *n*_*C*_ cooperators, *n*_*P*_ pool punishers, and *n*_*O*_ opportunists, such that

(A.1) $$n_{L}+n_{D}+n_{C}+n_{P}+n_{O}=n-1.$$

We need to distinguish three possible cases:

1. All other group members are loners (*n*_*L*_ = *n* −1); in that case the focal player becomes a loner automatically, yielding the payoff *σ*.
2. The group contains at least one other member that participates in the public goods game, but the group fails to establish a punishment institution ($n_{L}\leq n-2$, *n*_*P*_=0, and the focal player is not a pool punisher); in that case only cooperators contribute to the public good.
3. The group contains at least one other member that participates, and a punishment institution is established ($n_{L}\leq n-2$, and either the focal player is a punisher, or $n_{P}>0$); in that case cooperators, opportunists, and pool punishers contribute to the public good, and defectors are punished.

As groups are formed by randomly drawing *n*−1 co-players from the population, this distinction leads to the following formula for the expected payoff of a defector,

(A.2) πD=(NLn−1)(N−1n−1)·σ+∑nL≤n−2nP=0(NLnL)(ND−1nD)(NCnC)(NPnP)(NOnO)(N−1n−1)rc·nCn−nL−1+∑nL≤n−2,nP>0(NLnL)(ND−1nD)(NCnC)(NPnP)(NOnO)(N−1n−1)(rc·(nC+nO+nP)n−nL−1−β),

where the sum is taken over all possible group compositions $n_{L},\;n_{D},\;n_{C},\;n_{P},\;n_{O}$ subject to condition (A.1). Similarly, the expected payoffs of the other strategies are given by

(A.3) πC=(NLn−1)(N−1n−1)·σ+∑nL≤n−2nP=0(NLnL)(NDnD)(NC−1nC)(NPnP)(NOnO)(N−1n−1)(rc·nCn−nL−1−c)+∑nL≤n−2,nP>0(NLnL)(NDnD)(NC−1nC)(NPnP)(NOnO)(N−1n−1)(rc·(nC+nO+nP)n−nL−1−c),

(A.4) $$\pi_{P}=\frac{\left(\begin{array}{c} N_{L}\\ n-1 \end{array}\right)}{\left(\begin{array}{c} N-1\\ n-1 \end{array}\right)}\cdot \sigma +\underset{n_{L}\leq n-2}{\sum }\frac{\left(\begin{array}{c} N_{L}\\ n_{L} \end{array})(\begin{array}{c} N_{D}\\ n_{D} \end{array})(\begin{array}{c} N_{C}\\ n_{C} \end{array})(\begin{array}{c} N_{P}-1\\ n_{P} \end{array})(\begin{array}{c} N_{O}\\ n_{O} \end{array}\right)}{\left(\begin{array}{c} N-1\\ n-1 \end{array}\right)}(\frac{\mathrm{rc}\cdot (n_{C}+n_{O}+n_{P})}{n-n_{L}-1}-c-\gamma ),$$

and

(A.5) πO=(NLn−1)(N−1n−1)·σ+∑nL≤n−2nP=0(NLnL)(NDnD)(NCnC)(NPnP)(NO−1nO)(N−1n−1)rc·nCn−nL−1+∑nL≤n−2,nP>0(NLnL)(NDnD)(NCnC)(NPnP)(NO−1nO)(N−1n−1)(rc·(nC+nO+nP)n−nL−1−c).

Using the properties of multinomial distributions, one can simplify these expressions. A somewhat laborious computation confirms that the payoffs can be written as in Eq. (1). For a more detailed derivation, we refer to Schoenmakers (2013).
